# Improved Contact Predictions Using the Recognition of Protein Like Contact Patterns

**DOI:** 10.1371/journal.pcbi.1003889

**Published:** 2014-11-06

**Authors:** Marcin J. Skwark, Daniele Raimondi, Mirco Michel, Arne Elofsson

**Affiliations:** 1 Department of Biochemistry and Biophysics, Stockholm University, Stockholm, Sweden; 2 Science for Life Laboratory, Stockholm University, Solna, Sweden; 3 Department of Information and Computer Science, Aalto University, Aalto, Finland; 4 Interuniversity Institute of Bioinformatics in Brussels, ULB-VUB, La Plaine Campus, Triomflaan, Brussels, Belgium; Fudan University, China

## Abstract

Given sufficient large protein families, and using a global statistical inference approach, it is possible to obtain sufficient accuracy in protein residue contact predictions to predict the structure of many proteins. However, these approaches do not consider the fact that the contacts in a protein are neither randomly, nor independently distributed, but actually follow precise rules governed by the structure of the protein and thus are interdependent. Here, we present PconsC2, a novel method that uses a deep learning approach to identify protein-like contact patterns to improve contact predictions. A substantial enhancement can be seen for all contacts independently on the number of aligned sequences, residue separation or secondary structure type, but is largest for *β*-sheet containing proteins. In addition to being superior to earlier methods based on statistical inferences, in comparison to state of the art methods using machine learning, PconsC2 is superior for families with more than 100 effective sequence homologs. The improved contact prediction enables improved structure prediction.

## Author Summary

Here, we introduce a novel protein contact prediction method PconsC2 that, to the best of our knowledge, outperforms earlier methods. PconsC2 is based on our earlier method, PconsC, as it utilizes the same set of contact predictions from plmDCA and PSICOV. However, in contrast to PconsC, where each residue pair is analysed independently, the initial predictions are analysed in context of neighbouring residue pairs using a deep learning approach, inspired by earlier work. We find that for each layer the deep learning procedure improves the predictions. At the end, after five layers of deep learning and inclusion of a few extra features provides the best performance. An improvement can be seen for all types of proteins, independent on length, number of homologous sequences and structural class. However, the improvement is largest for *β*-sheet containing proteins. Most importantly the improvement brings for the first time sufficiently accurate predictions to some protein families with less than 1000 homologous sequences. PconsC2 outperforms as well state of the art machine learning based predictors for protein families larger than 100 effective sequences. PconsC2 is licensed under the GNU General Public License v3 and freely available from http://c2.pcons.net/.

This is a *PLOS Computational Biology* Methods article.

## Introduction

De novo protein structure prediction is a long-standing problem in protein bioinformatics. For many years the focus was to develop methods that accurately describe the free energy landscape of a protein and then use a search strategy to identify the conformation with the lowest free energy. Using this strategy, methods based on fragment-based assembly of proteins can sometimes produce surprisingly accurate models, in particular for small *α*-helical proteins [1]-[3]. In addition, molecular dynamics simulations can also accurately predict the structure of small fast folding proteins [4]. However, it is also clear that these methods are still not generally applicable to large-scale prediction of protein structures at high accuracy [5], [6]. An alternative approach is to first predict which residues interact and then use these interactions to predict the structure of the protein [7].

Knowing which residues in a protein interact with each other provides sufficient information to predict the structure of a protein [8], [9]. However, until recently, contact predictions were not sufficiently accurate to significantly aid protein structure predictions [10]. The most successful methods for contact predictions are based on identifying correlated mutations between pairs of residues [11]. The introduction of predictors using global models, inferring couplings between residues from the observed correlations, significantly increases the accuracy of co-variation based methods [12]-[16]. For accurate prediction these global contact prediction methods depend on accurate multiple sequence alignments of thousand or even more homologous protein sequences, using alignments from methods such as HHblits [17] and jackhmmer [18]. It has been shown that these global contact prediction methods actually provide sufficient information for predicting the structure of proteins belonging to large protein families [7], [19], [20].

In addition to utilising correlated mutations, earlier contact prediction methods used various machine learning approaches to improve the predictions [21]. In the first generation of these predictors every residue pair was considered independent from all other pairs. Here, features such as the amino acid type and conservation provided some improvement over only using the correlated mutations. In recent studies more complex machine learning algorithms have been used. These algorithms try to fully utilize the information, that can be inferred from the structure of a contact map using advanced machine learning methods including deep learning approaches [22]-[26] or constraint satisfaction of rules describing protein like contacts maps using linear programming [27].

Deep learning is a sub-field of machine learning in which multiple layers of non-linear processing are used to learn nonlinear mappings and abstract complex features through the subsequent layers of the deep architecture, thereby obtaining a hierarchical abstraction of the input data [28]. The techniques falling under the collective name of deep learning, thanks to their ability to define higher-level concepts by learning a feature hierarchy, can efficiently handle some typical artificial intelligence problems including image and object recognition, natural language processing and computer vision [29]. In general, deep learning is very useful in structured learning problems including protein contact predictions [30]. To our knowledge, deep learning has not earlier been combined with the improved contact prediction obtained from statistical inference contact prediction methods. Furthermore, the method described here is different from earlier deep-learning approaches: our implementation is based on a feed-forward stack of random forests learners and not layers of Neural Networks, as in earlier implementations. Further higher-level abstraction of the data is geared towards the recognition of secondary structure visual patterns in contact maps and to iteratively refine the initial predictions.

**Figure 1:**
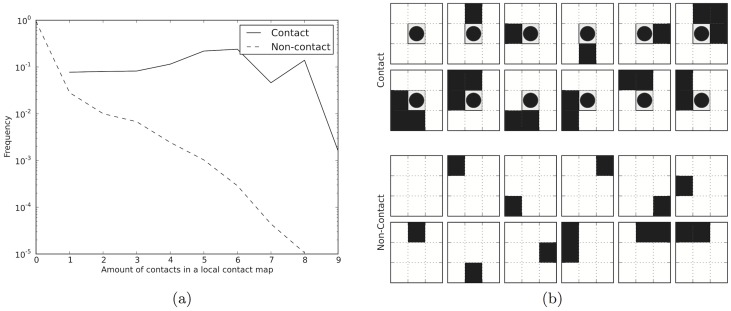
a) Relative frequencies of the number of contacts found in a 3×3contact map. Solid line represents the cases where a contact is present in the central position and the dashed line where the central position is a non-contact. The X-axis shows the number of contacts within the window, while the Y-axis shows the frequency of all contact maps with this number of contacts. b) Illustration of the twelve most frequent 3×3 contact maps when the central pair is a contact (marked with a circle) or a non-contact. In both figures the data is derived from a randomly selected subset of PDB and contacts are defined as elsewhere in the paper, that is C-*β*-C-*β* distance less than 8 Å.

The method is based on the fact that in proteins residue-residue contacts are often found in proximity of other contacts, while a non-contact is often found next to other non-contacts. In a 3×3 contact matrix with either a contact or a non-contact in the central position the frequency of contacts in the other positions differs significantly, see Figure 1. When a non-contact is present, the most frequent contact maps are the ones with none or only one contact present in the matrix. In contrast when a contact is present in the central position already the fifth most common contact map has three additional contacts and contacts maps with many contacts are more frequent, see Figure 1. Further, 98% of long-range contacting residues, compared to 30% for non-contacting pairs, are not isolated, i.e. contacts are found in the proximity of other contacts [26].

Moreover, the distribution of contacts follows characteristic patterns and recurring ones can be visually recognised and therefore used for constraining the prediction of nearby contacts. For instance if residues *i* and *j* in two *α*-helices are in contact, residues 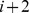 and 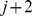 most likely will not be in contact. Contrary, a contact between residues *i* and *j* in two parallel *β*-strands implies strongly that the neighbouring residues, 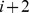 and 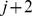, also are in contact.

Here, we show that a deep learning pattern recognition-based approach improves the accuracy significantly for all types of proteins, residue separations and numbers of homologous sequences. The obtained improvement is largest for *β*-sheet containing proteins, but exist for all type of proteins. Further, in comparison to state of the art other machine learning based methods contact prediction methods PconsC2 is superior for proteins with more than 100 effective sequence homologs.

## Results

**Figure 2:**
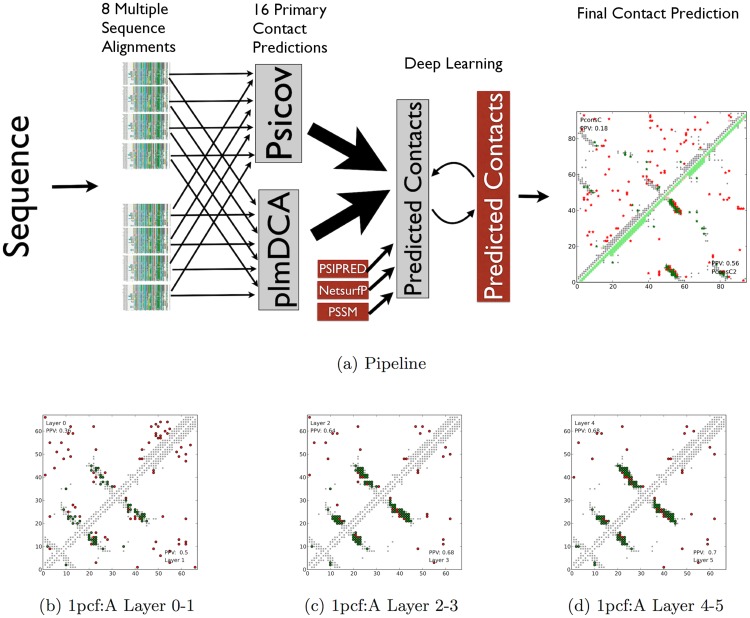
a) Overview of the PconsC and PconsC2 pipelines. For a query sequence; HHblits and jackhmmer are used to produce in total 8 multiple sequence alignments. In total 16 different contact maps are then produced using PSICOV and plmDCA. These are then fed into two different machine learning protocols, PconsC and PconsC2. In PconsC2 additional features are added and five layers of deep learning is applied. Predictions from PconsC and PconsC2 are shown to the right. b–d) An example on how the deep learning procedure used in PconsC2 improves predictions for the protein 1pcf:A. Each upper or lower triangle depicts the top *L* long-range predictions in a contact map predicted at one layer. Green dots represent correct predictions and red wrong predictions, while grey depicts true contacts. The values in the corners of the contact maps represent the fraction of correctly predicted contacts (PPV) within the top *L* long-range predictions. In this example the number of correct predictions doubles from the first to the last layer.

We recently developed PconsC, an ensemble method reconciling predictions across different contact inference methods and a range of multiple sequence alignments [31]. PconsC as well as PconsC2 are based on in total 16 contact predictions using a combination of HHblits [17] and jackhmmer [18] alignments with contact predictions from PSICOV [32] and plmDCA [33], see Figure 2. One limitation of PconsC, as well as all other global contact prediction methods, is that the processing of contacts is performed in isolation without considering nearby contacts. However, it was recently shown that such information could be utilised to improve contact predictions [30], [34]. Here, we present PconsC2 a deep learning based approach that considers nearby contacts and iteratively improves the contact predictions used in PconsC.

The development of PconsC2 was performed using five-fold cross-validation on 150 proteins reported in the PSICOV paper [32]. Further testing and analysis performance was performed using 383 proteins not related to the training set and on all proteins from CASP10 [35]. The general features of the three sets are described in Table 1. Two notable differences between the training set and the other sets are that some protein families in the test set have fewer homologous sequences and they neither are all strictly single domain non-interacting proteins.

**Table 1:** Properties of the three datasets.

	PSICOV	New	CASP10
Entries	150	383	114
Median 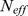	1029	165	1224
Median length	143 AA	161 AA	216 AA
Part of complex	9%	70%	50%
Multi-domain	0%	10%	22%

Median 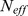 is the median number of effective sequences, computed by clustering sequences with identity above a pre-computed threshold. Part of complex is the fraction of proteins that are part of a complex according to PISA [49]. Multi-domain is the fraction of proteins containing more than one Pfam domain [50]. Median resolution is the median resolution of crystal structures, where numbers in brackets indicate fraction of structures from NMR.

### PconsC can be improved by using deep learning

**Figure 3:**
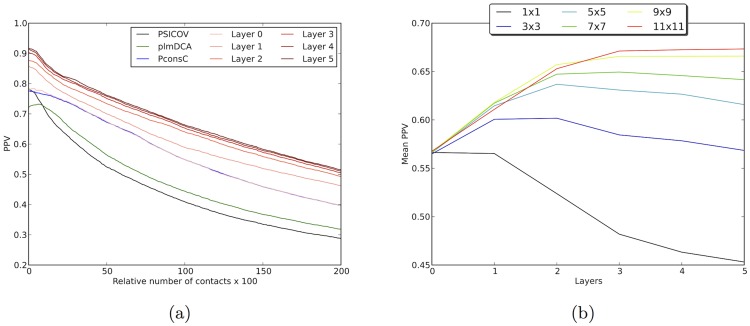
a) ROC plot depicting the PPV values for different predictors. The x-axis represents the number of contact predictions in relationship to the length of the protein. At 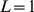 one prediction is included for each residue in each protein. The baselines are predictions from PSICOV (black), plmDCA (green) and PconsC (blue). Predictions from different layers in the deep learning procedure during training of PconsC2 are shown in red. The prediction at the first layer overlaps almost perfectly with prediction from PconsC. b) Impact of different sizes of the receptive field on prediction precision, measured at 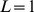 contacts. The X-axis represents each layer in the deep learning procedure.

First, we examined if a deep learning approach improves predictions using identical inputs as in PconsC [31]. PconsC and PconsC2 both use in total 16 predictions from PSICOV [32] and plmDCA [33] created from 8 different alignments. In the first layer, 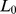, the prediction yields basically the same performances as PconsC, but is substantially better than the individual predictions from PSICOV or plmDCA, see Figure 3a. The small difference in performance to PconsC is due to the fact that PconsC was trained on the closest distance between any two atoms in a residue and not the C*β*-C*β* contacts used here, and due to differences in the training, as well as inherent noise in the training of the underlying machine learning method.

In layers 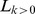 the predictions performed in the previous layer are taken into consideration in the *receptive field*, providing information about the neighbourhood of predicted contacts around each residue pair. Already using a *receptive field* of the size 3×3 provides some improvement, see Figure 3b, but the improvement is more substantial for window sizes up to 11×11, which is therefore used below. It is notable, that the major improvements in the prediction accuracy appears already on the first two layers of deep learning, gradually levelling off at the subsequent ones, see Figure 3a. Using too many layers, the architecture becomes over-trained and starts to recognise and reconstruct the visual patterns of secondary structures, exaggerating the clustering of the predicted contacts, obtaining predictions without biological or structural meaning.

Using a window of 11×11 and five layers of deep learning and evaluating the predictions at one prediction per residue (L = 1), a common cutoff used in earlier studies, the relative improvement between first and last layer in PPV is 54% (18% in terms of absolute PPV), see Table 2. In Figure 2 it can be seen that the deep learning procedure efficiently filters out sporadic isolated predictions, while increasing the amount of predicted contacts between secondary structure elements.

**Table 2:** PPV using different inputs features.

Features	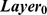	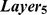
16 Predictions	0.57	0.67
16 Predictions + Separation	0.59	0.68
16 Predictions + SS	0.57	0.70
16 Predictions + PSSM	0.59	0.70
16 Predictions + RSA	0.59	0.71
16 Predictions + all features	0.61	0.73

Impact of different feature combinations on PPV values for first and fifth layer of deep learning.

### Further improvement using additional features

Next, we examined if it was possible to further improve the accuracy by including additional features. Inspired by current literature, we included four different features: Separation of contacting residues in the sequence (Separation), predicted secondary structures of residues surrounding the potentially contacting residues (SS), predicted solvent accessible surface area (RSA) and sequence profiles in the form of PSSMs (PSSM). All features improve the accuracy with a few percentage points both at the first and final layer, see Table 2. An additional small improvement can be obtained when combining all features. In total the improvement is 7–8% at the last layer in the deep learning procedure, see Table 2. The final version of PconsC2 uses all features and the predictions are further analysed below using the independent dataset of 383 proteins.

## Discussion

To further evaluate the performance of PconsC2 to other methods we have used three different datasets, see Table 1; First the 150 proteins from the PSICOV set used for training, secondly a completely independent dataset of 383 proteins not homologous to any protein in the training set and finally all proteins from CASP10. In the independent set the average number of homologous sequences is lower and therefore the average performance is lower as well, but the relative increase in performance from the training set is maintained. The performance analysis is done using predictions of one long-range contact per residue (L = 1, with sequence separation of at least 5 amino acids) for each protein in the test set. This is a common way to evaluate contact prediction methods and it has been shown that the relative performance differences are quite independent on the number of contacts tested [10]. At 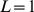 the positive predictive values, PPV, precision, increases from 0.25 for the best single methods (plmDCA) to 0.29 for PconsC and to 0.44 for PconsC2.

### PconsC2 improves predictions at all sequence separations

**Figure 4:**
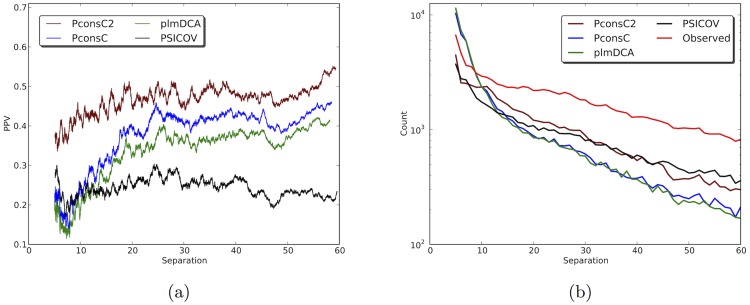
a) Performance of PconsC2 at different sequence separations compared to PconsC, plmDCA and PSICOV, considering top *L* contacts per protein. Curves are smoothed with a rolling average window of 5 residues. b) Number of contacts predicted at different sequence separations. The read line represent the distribution of observed contacts in the dataset, normalised in so that the total number of contacts is identical to the number of predicted contacts.

Contacts at different separation provide different types of information and the underlying contact prediction methods; plmDCA and PSICOV, behave quite differently in this aspect. Both methods predict a lower fraction of long-range contacts than observed in proteins, but PSICOV predicts more long-range contacts among its top-ranked predictions than plmDCA, see Figure 4. However, the accuracy of these long-range predictions is lower. And the reverse is true for short-range contacts (separated by less than 10 residues), here PSICOV predicts fewer but more accurate contacts. The distribution of predictions from PconsC is quite similar to the distribution from plmDCA, just with a higher accuracy.

The separation distribution for the top ranked contacts for PconsC2 resembles the distribution of contacts from PSICOV. However, the accuracy for contacts predicted by PconsC2 is higher and not strongly dependent on residue separation, see Figure 4. Both the increased accuracy of short-range contacts and in particular the increased number of long-range contacts should be useful for structure prediction, as accurately predicted long-range contacts contain more structural information.

In a comparison to other contact prediction methods PconsC2 exhibits greater accuracy at all sequence separations, but the performance improvement is most prominent for the contacts with sequence separation of 12 amino acids or more, see Table 3. The improved accuracy is evident independent on the number of residues analysed, while the remaining results are consistent with performances reported previously in the literature [27].

**Table 3:** PPV values for contacts at different separations.

Range	PconsC2	PconsC	PSICOV	plmDCA	MI	CMIr	CMAPpro	PhyCMAP
Short (6–12)	0.53	0.34	0.30	0.31	0.08	0.16	0.49	0.50
Medium (12–24)	0.56	0.41	0.33	0.37	0.09	0.20	0.44	0.44
Long (24+)	0.55	0.46	0.37	0.41	0.09	0.19	0.37	0.35

Prediction accuracy at particular sequence separations taking into account L/10 predicted contacts in the class.

### PconsC2 improvement is largest for *β*-sheets

The interaction patterns between different secondary structure elements vary significantly. In particular, parallel and anti-parallel *β*-sheets are easily recognisable in a contact map because of their dense visual patterns. On the other hand contacts between *β*-sheets are primarily mediated through backbone hydrogen bonds, suggesting that these might not be under the same co-evolutionary pressure as contacts mediated by sidechains, that is they might be harder to identify using methods based on co-variation of the sidechains. To analyse the effect of secondary structure, all proteins in the independent dataset were classified into their fold classes according to CATH [36].

**Figure 5:**
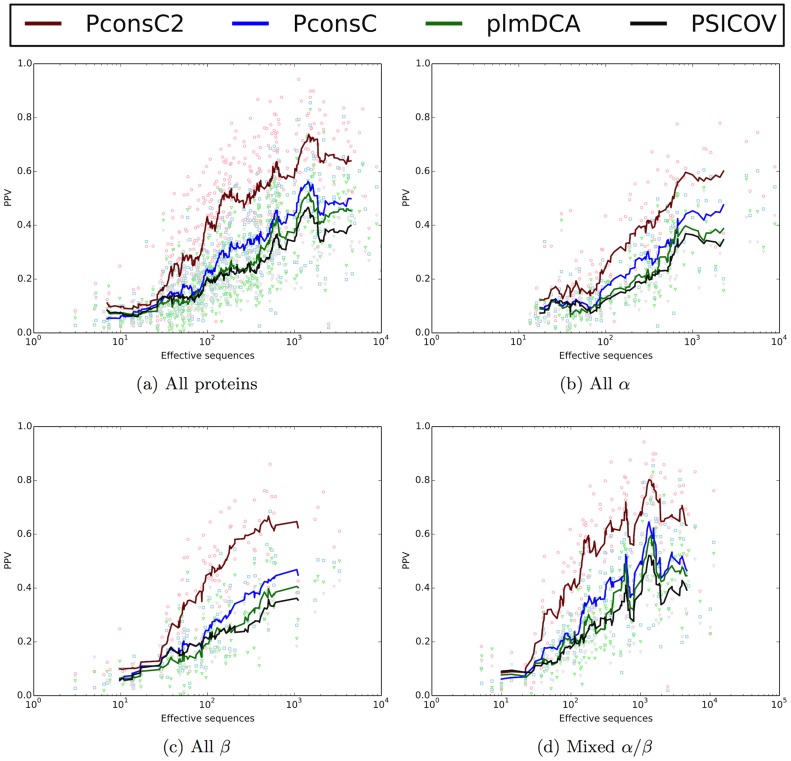
Positive predictive value plotted versus efficient number of sequences for predictions made by PSICOV, plmDCA, PconsC and PconsC2, considering top 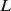 contacts per protein target. Line: running average with frame size 20. (a) All proteins, (b) all-*α*-helical proteins (c) all- *β*-sheet proteins and (d) mixed *α/β*-proteins.

In Table 4 and Figure 5 it can be seen that the improvement obtained by PconsC2 is largest for all *β* proteins and least for all *α* proteins. The overall performance is highest for the mixed *α*/*β* proteins followed by all *β* proteins.

**Table 4:** PPV values at 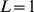 for different protein structural classes.

Structural class	No	PSICOV	plmDCA	PconsC	PconsC2
all *α*	119	0.18	0.20	0.24	0.33
*α/β*	159	0.27	0.31	0.36	0.53
all *β*	98	0.21	0.21	0.25	0.40
few sec.str.	7	0.24	0.29	0.35	0.47
All	383	0.23	0.25	0.29	0.44

In Table 5 individual contacts between residues in different secondary structure elements are analysed. For all types of secondary structures PconsC2 produces more correct predictions than the other methods. However, due to its design, PconsC2 tends to over-emphasise contacts between secondary structures in comparison to the contacts involving loop regions. While the dataset contains only 22% of 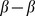 long-range contacts, PconsC2 assigns 34% of its top-ranked predictions to this type of interaction. PconsC2 predicts more than twice as many 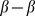 contacts than any other method, while still maintaining a slightly higher accuracy. A similar but less pronounced effect can be observed for the 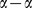 contacts, where PconsC2 assigns 15% of its top ranked contacts to this class, although contacts comprise only 11% of the observed contacts. In contrast, contacts involving loop residues are less frequent among top ranked contacts. In particular contacts between loops and another secondary structure are only rarely predicted. However, thanks to the increased accuracy the number of correctly identified contacts is actually similar, or higher, for PconsC2 also for loop-loop contacts, highlighting the ability of PconsC2 to filter out false predictions.

**Table 5:** PPV values at 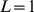 for contacts between different secondary structure classes.

Structural category	Real	PSICOV	plmDCA	PconsC	PconsC2
*α* - *α*	11%	0.23 (15%)	0.22 (17%)	0.28 (16%)	0.40 (15%)
*α* - *β*	8%	0.31 (9%)	0.46 (7%)	0.48 (8%)	0.54 (7%)
*α* - loop	16%	0.18 (21%)	0.22 (21%)	0.27 (21%)	0.37 (13%)
*β* - *β*	22%	0.41 (13%)	0.51 (12%)	0.52 (13%)	0.57 (34%)
*β* - loop	22%	0.20 (23%)	0.22 (21%)	0.26 (22%)	0.46 (16%)
loop -loop	21%	0.19 (18%)	0.17 (22%)	0.24 (19%)	0.30 (16%)
ALL		0.24	0.26	0.31	0.46

Accuracy in different structural categories considering top L contacts per protein. Percentage shows the relative fraction of predicted contacts in that category.

### PconsC2 produce accurate contact predictions for smaller families

The success of statistical inference contact prediction methods, such as PSICOV and plmDCA, depends strongly on the availability of thousands of aligned sequences. Today the genome projects have already provided a sufficient number of homologous sequences for more than one hundred protein domain families [37]. In the future increased sequencing efforts will eventually expand the range of proteins for which these methods are applicable, thus increasing the number of potential prediction targets. However, as protein domain family sizes follow a power-law distribution [38], the vast majority of protein families may never reach the thousands of members that are needed for successful predictions. Therefore, it is of great importance to increase the accuracy of contact predictions for smaller protein families.

We examined to what extent PconsC2 could increase the performance for smaller families. Due to the way the input alignments are constructed, the total count of sequences in the alignment is not the most suitable metric for the information content, as a high number of close homologs do not provide as much co-variation as comparable number of more distantly related proteins. As suggested before a better measure is to use the number of efficient sequences to take redundancy into account [32].

In Figure 5 it can be seen that PconsC2 maintains the superior predictive performance throughout the whole set of alignments, independently on the size of the protein families. It attains an average prediction precision of 0.40 for alignments as small as 100 efficient sequences. This is on par with the PPV values obtained for protein families with thousands of sequences using the other methods. In particular for all *β*-proteins the plateau performance is reached at a lower number of sequences than for earlier methods. Although the average PPV for PconsC2, as well as earlier statistical inference methods, depend strongly on the number of efficient sequences in the alignment it is clear that PPV values of 0.5 or even higher are reached even for protein families with fewer than 100 effective sequences, see Figure 5. If this performance could be pushed to all protein families of this size the usefulness of contact prediction methods for structure prediction would increase significantly.

### PconsC2 performs better than earlier predictors

In addition we compare PconsC2 to a set of alternative contact prediction methods, including mutual information (MI) and MI with phylogenetic correction (CMIr), and two recent machine learning based methods, PhyCMAP and CMAPpro.

**Figure 6:**
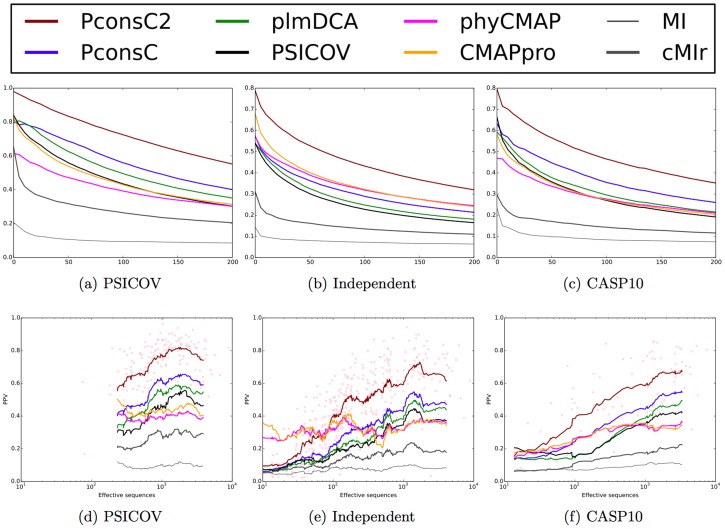
(a–c) ROC plot depicting the PPV values for different predictors. The x-axis represents the number of contacts prediction in relationship to the length of the protein. At L = 1 on average one prediction is included for each residue in a protein. (a) Performance on the PSICOV set, (b) Performance on the new dataset, (c) Performance on the CASP10 dataset. (d–f) Positive predictive value plotted versus efficient number of sequences for predictions considering top 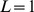 contacts per protein. The lines show a running average and the red dots individual predictions by PconsC2.

In all three datasets PconsC2 shows a higher performance than all the other methods, see Figure 6a-c and S1. At one prediction per residue, 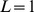, the PPV values of PconsC2 ranges from 0.75, in the PSICOV set to 0.5 in the independent dataset. In comparison plmDCA PPV values range from 0.5 to 0.25 and CMAPpro has PPV values of approximately 0.45 to 0.3. In all sets the PPV values are at least 0.1 units higher for PconsC2 than for the best of the other methods. The improvement exist for all sequence separations, see Table 3.

Next, we compared the performance of the different methods given the number of effective sequences in the alignments. For PconsC and PconsC2, which use several multiple alignments, we used HHblits with an E-value cutoff 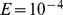 as an estimation of the number of sequences. As has been reported before methods that use statistical inference show a strong dependency on family size with a strong increase in performance between 100 and 1000 effective sequences [39]. In contrast the performance of the machine learning methods is rather unaffected by the family size. As a consequence of this machine learning methods outperform all methods for families up to 100 effective sequences, and all methods except PconsC2 for families up to a few hundred effective sequences. However, these methods are clearly outperformed by all statistical inferences methods for large families.

There is a difference in performance on the PSICOV set versus the other two dataset given the same the number of effective, see Figure 6d-f and S1. This difference is smaller for the machine learning based methods than for the correlated mutation based methods. In addition to having on average larger families the PSICOV dataset contains strictly single domain proteins, while the other sets contain both single and multi-domain proteins and proteins that are parts of large complexes, see Table 1. The drop in performance is most likely caused by non-conserved domain-domain or protein-protein interactions. Further, accurate multiple sequence alignments of large multi-domain protein families is difficult.

### Good and bad examples

**Figure 7:**
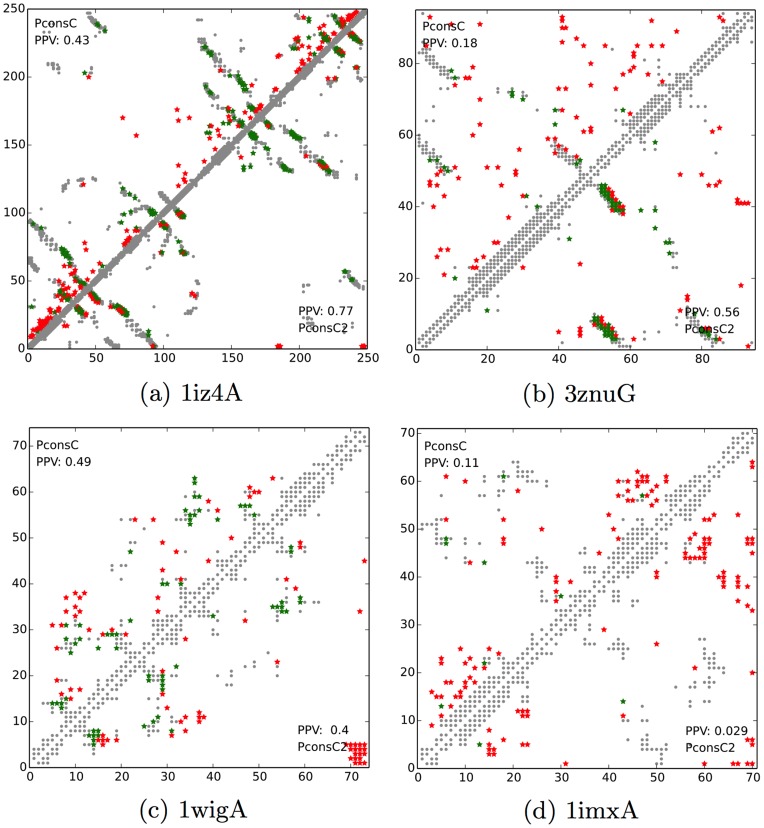
Contact maps for four proteins; (a) 1z4A and (b) 3znuG, (c) 1wigA and (d) 1imxA. The upper-left triangle depicting the contact map predicted by PconsC, and lower-right triangle by PconsC2. Grey dots indicate the real, observed contacts in PDB structures, while coloured ones depict the contacts predicted by respective methods. Here red represent wrong predictions and green correct ones. The values in the corners of the contact maps represent the fraction of correctly predicted contacts within the top 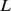 long-range predictions.

To highlight the behaviour of PconsC2 in comparison to PconsC we selected a few examples to highlight when PconsC2 significantly improves the predictions, see Figure 7.

The first example, *P. furiosus* DNA polymerase sliding clamp (1iz4A), contains two DNA clamp domains, which are in a predominantly *β* conformation. In comparison to PconsC, PconsC2 filters out spurious short- and medium-range predictions, located at the domain termini. In addition, PconsC2 increases the predicted contact density in the *β*-sheet regions of both domains.

The second example, *R. opacus 1CP* chloromuconolactone dehalogenase ClcF (3znuG), is a homo-10-meric protein. Here, we only predict contacts within one chain and the prediction performance of PconsC is poorer (PPV = 0.18) than of the constituting methods (plmDCA, PPV = 0.39 and PSICOV PPV = 0.29). This is due to a limited overlap between the individual predictions, resulting in a nearly random distribution of predicted contacts. However, the use of additional information, as well as pattern recognition in PconsC2 allows for reconciling the conflicting predictions and attaining a better prediction than any of the individual methods (PPV = 0.56).

Obviously, there also exist proteins for which the PconsC2 methodology does not work and occasionally PconsC2 performs worse than PconsC. In general these cases can be divided into two categories. PconsC2 tends to perform badly, when the underlying predictors provide conflicting or low quality information. PconsC2 also sometimes fails when the deep learning methodology introduces artefacts into the contact map. We have paid close attention to reducing the amount of artefacts introduced by deep learning, which is one of the main reasons behind limiting the number of prediction layers.

The first example of PconsC2 failing with respect to PconsC is the LIM domain of RSGI RUH-019 (1wigA), which is a relatively short (73 amino acids) protein that contains few secondary structures. Here, PconsC2 produces a spurious cluster of predictions between the N- and C-termini, reducing the PPV from 0.49 to 0.40, see see Figure 7c.

The second example is the human insulin-like growth factor 1A (1imxA), for which neither PconsC nor PconsC2 make usable predictions. It is worth noting, that the contact patterns predicted by both approaches are quite different, with PconsC predicted contacts being more uniformly distributed along the sequence, while the PconsC2 attempts to create incorrect interactions between secondary structure elements, see Figure 7d.

### Using PconsC2 improves protein modelling

One of the ultimate goals of contact prediction is to facilitate *ab-initio* protein structure prediction. To test if the improved contact predictions from PconsC2 indeed are more useful for protein modelling, we built models for all proteins in the independent test set using both PconsC and PconsC2 predicted contacts, using the PconsFold pipeline [40]. Models constructed using contacts predicted by PconsC2 are on average 9% better in terms of TM-score [41], than those using contacts predicted by PconsC, see Table 6. As expected from the PPV analysis the improvement is largest for *β*-sheet containing proteins, see Table 6.

**Table 6:** Average TM-score of models generated with contacts from PconsC or PconsC2.

Structural class	No	PconsC	PconsC2
all *α*	119	0.36	0.39
*α/β*	159	0.38	0.41
all *β*	98	0.26	0.29
few sec.str.	7	0.33	0.35
All	383	0.34	0.37

**Figure 8:**
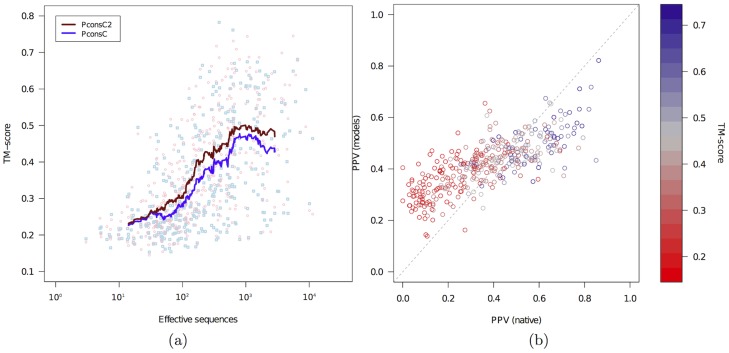
(a) Comparison of the quality of models generated using the PconsFold pipeline [40] and contacts predicted by either PconsC or PconsC2. In PconsFold, Rosetta [2] is used together with predicted contacts from PconsC (blue) or PconsC2 (red). Each dot represents one model and the lines show the running average. The average TM-score for models generated with PconsC contacts is 0.34 vs. 0.37 for models generated using PconsC2. (b) Comparison of agreement with predicted contacts for the native structure and the top-ranked model. The colours of each dot represent the TM-score of the model. Dots below the diagonal line indicates that the model agrees worse with predicted contacts than the native structure, that is it should be possible to obtain better models given a more efficient modelling procedure.

The average quality increases with the effective number of sequences in the protein family, see Figure 8. However, the variation is also rather large, that is for some small families good models are generated, while for some large families the models are not optimal. The average improvement obtained by PconsC2 is quite constant for families with more than 50 effective sequences.

One likely reason why the improvement of model qualities not is larger might be that our folding protocol cannot fully utilize the improved contacts. In Figure 8b it can be seen that for many of the proteins with best PPV values the predicted contacts are much better fulfilled by the native structure than by the top ranked model, that is the contact information is not fully utilised in our folding protocol, i.e. there is room for improvement.

### Final summary

Earlier statistical inference co-evolution based contact prediction methods assume independence between the couplings calculated among pairs of residues, but contacts in a protein contact map are neither randomly, nor independently distributed. Their distribution clearly follows characteristic patterns for certain types of interactions, e.g. hydrogen bonding ladders between *β*-sheets, which can be recognised. When a hypothetical contact is predicted between residue *i* and *j* it is intrinsically conditioned that the contact likelihood of – at least – the pairs of residues close to *i*, *j* are affected. Such rules have been used in other contact prediction methods, not using the statistical inference approach, either by satisfying specific constraints [27] or be using a deep learning approach [25], [26]. Here, we have developed a novel method, PconsC2, which uses a deep learning approach taking this information into account to improve contact predictions.

PconsC2 improves the prediction for all proteins and all types of contacts, but the improvement is most significant for *β*-sheet containing proteins. Perhaps most importantly, already at 100 effective sequence homologs the average prediction accuracy from PconsC2 rivals the maximum accuracies obtained from earlier methods and for larger families PconsC2 outperforms all methods. However, other machine learning approaches outperform PconsC2 for smaller families indicating room for improvement. The fact that PconsC2 predicts a larger fraction of contacts separated by many residues contributes to that models produced using contacts from PconsC2 instead of PconsC are on average 9% better.

## Methods

There exist different possible definitions for residue contacts. The most commonly used are based on C*α*-C*α*, C*β*-C*β* or the closest heavy atom distances in sidechains. The relative improvement of PconsC2 is independent on the definition and therefore we chose to present only the results using the same contact definition as used in CASP (Critical Assessment of protein Structure Prediction), that is C*β*-C*β*, C*α* for Glycine, distance between the amino-acids _≤_8Å [42].

### Dataset

PconsC2 has been trained on a set of 150 non-large, single-domain proteins used for evaluation of PSICOV [32] and used for evaluation of PconsC [31]. The intermediate results have been obtained using this set, with 5-fold cross validation, with regard to CATH superfamilies. The datasets are available from Table S1 and https://c2.pcons.net/.

The final evaluation was conducted on a set of 383 proteins, which are not evolutionarily related to the proteins in the training set. This set was constructed starting from approximately 650 proteins of known structure, including proteins representing the most common folds. This set was then homology reduced to ensure that no protein in this set has detectable hits with E-value lower than 10^−3^ to any of the proteins in the training set or the proteins in testing set. Homology reduction was done by a jackhmmer search against UniRef100. Finally proteins with very few homologs and not compact were rejected. We considered proteins to be not compact enough if they have a small amount of long-range contacts, long intrinsic disordered regions, or large unstructured termini.

For the CASP10 datasets all protein sequences were downloaded from the CASP website. Comparisons were performed both against the predictions submitted to CASP and by running all predictors again, taking into account increases in databases' size etc. An overview of the three datasets is presented in Table 1.

#### Effective sequence

The number of sequences included in an alignment can be used as a rough indicator of the expected accuracy of the contact predictions. However, a better correlation is obtained when effective number of aligned sequences is used, i.e taking the redundancy into account. Here, we have used the same definition as used in the PSICOV article [32], but other definitions provide very similar results. The effective number of aligned sequences is calculated as follows; First, the mean sequence identity (MeanID) is calculated for all sequences and then a similarity threshold is computed as:
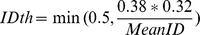



Then all sequences that have fewer than 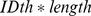 differences are counted as a single sequence for the purpose of computing the number of sequences in the alignment.

#### Comparison to other methods

For benchmarking, predictions from PconsC2 are compared with predictions from a number of other methods. These can basically be divided into three groups, mutual information methods that only use mutual information, global information methods that infer the observed mutual information from estimated direct interactions, and machine learning based methods that use mutual information as well as other information to predict contacts.

Here, Mutual Information (MI) and Corrected Mutual Information (CMIr) are calculated using a freely available package developed by Bahar [43], [44]. Here, the alignment refinement removes any rows that have more than 20% gaps. CMIr is calculated using the average product correction [45], which has been shown to improve residue contact predictions.

Further three statistical inference methods; plmDCA [33], PSICOV [32] and PconsC [31] were also included in the benchmark. Prediction for PSICOV and plmDCA were based on the HHblits alignments with an E-value cutoff 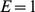, as this provided the best performance. As described elsewhere PconsC and PconsC2 use predictions from plmDCA and PSICOV as inputs to their machine learning methods.

Finally, we also tested three machine learning based contact predictors; PhyCMAP [27], CMAPpro [26] and DNcon [25]. PhyCMAP uses mutual information as well as other constrains to construct an objective function [27]. In addition it utilizes linear physical constraints describing how contacts can be related to each other in a protein. The objective function is then optimised given the constraints using integer linear programming. This results in a physically feasible contact map. In CMAPpro and DNcon mutual information and other features are used to predict an initial contact map, this is then refined using a deep learning approach [6]. Unfortunately, we did not manage to run all queries through DNcon website, but preliminary data for about 100 proteins did not indicate that it would perform better than CMAPpro or PhyCMAP. Therefore, at the end predictions were only compared with PhyCMAP, which was run locally, and CMAPpro, which was run through its website as a downloadable version is not available. It can be noted that although the performance of PconsC2 is superior to the other methods it is also significantly computationally more expensive.

### Deep learning

The machine learning architecture implemented in this paper is inspired by deep learning literature in the sense that it is a feed-forward stack of learners, in which each layer 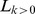 refines the predictions provided by the previous layer. Deep learning approaches are generally implemented through *deep* neural networks architectures (with many hidden layers of neurons) and are particularly useful in structured problems. It is because the hidden layers can learn and store complex *higher level* feature representations for the activation signals provided by the input layer, thus mimicking in a certain sense, the concept of high-level *concepts*. This behaviour can be extremely valuable for solving structured problems in which some high-level structure underlies the input data, such as image, speech and handwriting recognition. Deep learning architectures based on neural networks have some drawbacks: for example they are computationally demanding and require careful tuning [46]. In this work we chose to lower the complexity of the machine learning procedure and architecture while preserving the ability to *abstract* higher level features from the data (the secondary structure visual patterns in the contact maps). We implemented a multilayer feed-forward stack of random forest learners, but in principle any machine learning algorithm could be used in each layer; for this reason, during the description of the architecture, we refer with the generic term of “learner” to the algorithm used in each layer.

Let 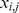 be the feature vector representing the residue pair 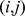. These features are taken in input by the first layer 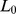 of the architecture, obtaining the predictions 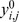. In the subsequent learning layers 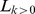, for each residue pair 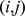 the input consist of the feature vector 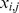 plus a subset of the predictions of the previous layer. This subset represents the probabilities of contacts in the neighbourhood of 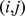, named the *receptive field* [30].

The approach described here is conceptually different from the one adopted by Di Lena et al. [30]. In particular, they used a 3-dimensional stack in which each layer is composed by many *positional* neural networks, one for each position 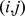 in the contact map. Our method is computationally less demanding as only one learner for each layer is used. In each layer the single learner handles all of the prediction of the entire contact maps regardless of the positions 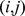. The learner can still abstract complex features, such as the secondary structure patterns, from the receptive field.

The architecture can be described with the following recurrent formalisation:

- Layer 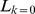: 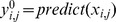

- Layers 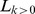: 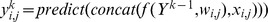

where 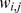 is the neighbourhood of 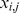 and 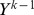 is the set containing all predictions 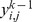 obtained from the layer 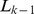. The function 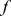 selects the receptive field and is defined as:


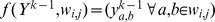
 with 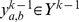.

Here, the receptive field is a square matrix of 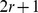 by 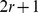 pairs representing the predicted probability of a contact for the neighbouring residue pairs.

### PconsC2 is using Random Forests

During the development of PconsC2, we observed certain inherent characteristics of input data and the contact prediction problem, which can be summarised in the following three points:

1. The contact prediction problem requires the elaboration, for each *L*-residue long protein, of a number of vectors in the order of 
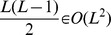
, that is the machine learning method must efficiently handle large sets of data.
2. The number of contacts increases almost linearly with protein length, but since the number of possible contacts increases quadratically, the contact density of a protein decreases with the inverse of the protein length. This means that the set of vectors for training is heavily unbalanced.
3. The dataset (consisting of predictions from the single coevolution based methods) is quite noisy and might contain artefacts, as certain strong couplings detected by these methods may have arisen for reasons other than intra-chain spatial proximity.

We found, in spirit of PconsC [31], that ensemble methods, in particular Random Forests, tend to result in most robust and accurate predictive models. This is likely due to the diversity among the learners, which allows for correcting the misclassifications of certain predictions.

In PconsC2 each layer of the deep learning architecture uses a Random Forest Classifier [47] with 100 trees and a minimum of 500 samples for any newly created leaf to avoid over fitting. These constraints have been introduced in order to build a forest with the greatest generalisation power possible.

### Receptive field

As described above, each layer 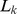 (with 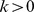) takes as input two different types of features, the *ordinary features* and the receptive field, concatenated. The ordinary features are identical for each residue pair in every layer, while the receptive field 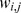 is a squared set of predictions centered in each couple of residues 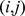, with an area of 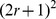 predictions obtained from the previous layer. As can be seen in Figure 3 the prediction performance increases with increasing size of the receptive field. We chose 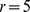, giving a field size of 11×11, as the best compromise between prediction precision and performance.

### Feature encoding scheme

PconsC2 uses the same combination of predictions from PSICOV [32] and plmDCA [33], as PconsC. The results have been obtained, using four different E-value cut-offs (10^−40^, 10^−10^, 10^−4^ and 1) to construct alignments by HHblits (from HHsuite 2.0.16) and jackhmmer from HMMER 3.0. The HHblits alignments used the bundled uniprot20_2013_03 database. Disabling both filtering the resulting MSA (-all parameter) and limiting the amount of sequences, that are allowed to pass the second pre-filter, as well as allowing for realigning all the hits in HHblits lead to the construction of the most information-rich and accurate alignment at cost of slightly increased running time. Jackhmmer alignments were constructed with UniRef100, based on UniProt 2013_06 release, with at most 5 rounds of iterative search and inclusion threshold (incE) set to the same value as the E-value threshold.

#### Additional input features

In addition to the 16 contact predictions we examined the possibility to use additional information in the form of sequence separation, PSSMs as well as predicted structural features.

Sequence separation is the distance between amino acids in sequence space, or differently formulated, the absolute value of difference between amino acid indices.

For each protein in the dataset, a sequence profile starting from the MSA obtained with HHblits with E-value cut-off _1_ was calculated. In the feature vector we represent each position *_i_* of the protein sequence with 21 real values, denoting the logarithm of ratio of frequency of an amino acid at this position to the background frequency of this amino acid in UniRef50. As the predictions take into account two positions in the sequence at the time, this results in 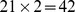 new dimensions in the feature vector. This feature is referred to as “PSSM” in Table 2.

The secondary structure prediction was calculated feeding PSIPRED with the alignment obtained by running HHblits with E-value cut-off 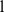 and filtered down to diversity 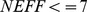 by *hhfilter*, as implemented in *addss.pl* script of HHsuite. The secondary structure prediction for each residue was encoded using a vector of probabilities for each of the structural classes (Helix, Strand, Coil). Here, we used a window of 9 residues (i-4, i+4) providing 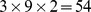 new dimensions, referring to this feature as “SS”.

Predicted relative solvent accessibility information calculated with NetSurfP [48] was also used. Best performance was obtained using window size of 9 residues, encoding relative surface accessibility, Z-fit score (reliability value), plus probabilities for three secondary structure classes as predicted by NetSurfP (Helix, Strand and Coil). This results in 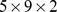 new dimensions in the feature vector, denoted as “RSA” in Table 2.

The secondary structure features predicted by NetSurfP and PSIPRED may seem redundant, though methods used are not the same. Predictions by PSIPRED come at nearly negligible computational cost, as they use pre-computed alignments, whereas NetSurfP predictions come “free” with predicted RSA and the inclusion of both provided a slight improvement.

In summary, the additional features are:

- PSICOV and plmDCA are, respectively, the 8 predictions from PSICOV and from plmDCA (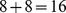 dimensions)
- Separation is 1 dimension denoting the distance between amino acids in the sequence space
- SS are the 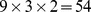 dimensions representing the predicted Secondary Structure
- PSSM are the 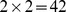 dimensions containing the sequence profiles calculated from HHblits alignment at E-value cut-off equal to 1
- RSA are the 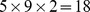 values used to represent the predicted relative surface accessibility, its confidence and predicted secondary structure.

### Availability

PconsC2 is licensed under the Gnu Public License. The source code is available from https://github.com/ElofssonLab/PconsC2/. However, to run it a rather substantial set of other programs are needed, including plmDCA, Matlab, PSICOV, HHblits and jackhmmer. Therefore, we also provide iso-images containing all necessary tools and databases. We do also provide a web-server at http://c2.pcons.net/. Unfortunately given the computational costs involved in running PconsC2 the capacity of the web-server is somewhat limited.

## Supporting Information

Figure S1Figure analogous to Figure 6c and f, depicting performance of discussed methods on CASP10 proteins, including additional methods. (a) ROC plot depicting the PPV values for different predictors on the CASP10 dataset. The x-axis represents the number of contacts prediction in relationship to the length of the protein. At L = 1 on average one prediction is included for each residue in a protein. Performance. (b) Positive predictive value plotted versus efficient number of sequences for predictions considering top 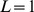 contacts per protein. The lines show a running average and the red dots individual predictions by PconsC2.(EPS)

Table S1 PDB IDs and sequences of the training- and testsets used in this study.
(TXT)
